# The Role of von Willebrand Factor Antigen in Predicting Survival of Patients with HBV-Related Cirrhosis

**DOI:** 10.1155/2022/9035971

**Published:** 2022-03-22

**Authors:** Youmin Pan, Renyong Guo, Yan Lv, Dawei Cui, Jue Xie

**Affiliations:** ^1^Department of Blood Transfusion, The First Affiliated Hospital, Zhejiang University School of Medicine, Hangzhou, China; ^2^Department of Laboratory Medicine, The First Affiliated Hospital, Zhejiang University School of Medicine, Key Laboratory of Clinical In Vitro Diagnostic Techniques of Zhejiang Province, Hangzhou, China

## Abstract

**Objective:**

The model for end-stage liver disease (MELD) scoring system cannot be used to assess the deterioration of patients with liver cirrhosis caused by infection and portal hypertension. Elevated von Willebrand factor antigen (vWF-Ag) in plasma is associated with portal pressure and complications in patients with liver cirrhosis. We aimed to evaluate whether the addition of vWF-Ag can improve the risk prediction ability of the MELD scoring system.

**Methods:**

A total of 228 patients with hepatitis B virus (HBV)-related liver cirrhosis were eligible for inclusion in this retrospective study. The vWF-Ag level was assessed by enzyme-linked immunosorbent assay (ELISA). The endpoint of this study was defined as the time to liver transplantation or death. Univariate and multivariate analyses were performed to assess the risk factors associated with transplant-free mortality. Receiver operating characteristic (ROC) curve analysis was used to assess potential discriminatory variables for transplant-free mortality.

**Results:**

During a median follow-up interval of 30.23 months, 124 patients (54.4%) reached the endpoint of this study. Patients who died or underwent liver transplantation had elevated levels of MELD and vWF-Ag. Moreover, vWF-Ag and MELD showed comparable predictive potential for transplant-free survival (area under the curve [AUC], vWF-Ag = 0.71; AUC, MELD = 0.73). Ultimately, vWF-Ag can significantly improve the predictive potential of MELD in determining transplant-free mortality (AUC, MELD-vWF-Ag = 0.79, *P* = 0.006).

**Conclusion:**

An elevated vWF-Ag level was independently associated with transplant-free mortality in patients with liver cirrhosis. The inclusion of vWF-Ag in the MELD scoring system can improve mortality predictions in patients with liver cirrhosis.

## 1. Introduction

Liver cirrhosis, a complication of chronic liver disease, is commonly caused by viral, alcoholic, or autoimmune hepatitis, as well as by other types of chronic or recurrent liver damage [1]. Patients with cirrhosis experience multiple changes in the haemostatic system, which lead to certain frequent complications, such as portal vein thrombosis and ascites, extensive damage to multiple organs, and high mortality [2, 3]. In these patients, prognostic indicators are needed to stratify care and assign specific treatments and liver transplantation. The model of end-stage liver disease (MELD) is widely used to predict the prognosis of patients with cirrhosis [4, 5]. However, the MELD score may not be able to predict patient outcomes, especially those with compensated cirrhosis and lower MELD scores [6, 7].

The von Willebrand factor (vWF) is a key component of the haemostatic system involved in primary haemostasis. It is a multifunctional large multimeric protein with multiple domains and harbours binding sites for collagen, platelet glycoprotein receptors, and coagulation factor VIII [8], mediating platelet adhesion to subendothelial collagen in the damaged vessel and indirectly carrying factor VIII by forming a noncovalent complex with it to bring it to injured vessels [9–11]. The function of vWF in primary haemostasis is located in the arterial and microcirculation, and it can contribute to thrombosis directly [8]. Previously, numerous studies have shown that patients have significantly elevated vWF-Ag levels compared to healthy controls because of overproduction from endothelial cells damaged by liver injury [12–15]. vWF-Ag is a multimeric glycoprotein synthesized in endothelial cells and megakaryocytes [16, 17] and is a biomarker of endothelial function. Patients with cirrhosis experience endothelial dysfunction in the hepatic vascular bed, which contributes to the release of vWF in activated endothelial cells; therefore, endothelial dysfunction is considered a major determinant of increased vWF-Ag levels [18–21].

Prasanna et al. revealed that the elevated vWF-Ag levels are correlated with organ failure and can predict in-hospital survival in acute-on-chronic liver failure patients [22]. Another study showed that vWF-Ag is a predictor of short-term mortality (1 week) in patients with acute-on-chronic liver failure and medium-term mortality (1–3 years) in patients with cirrhosis [23]. To our knowledge, no studies have yet focused on the role of vWF-Ag in predicting the long-term survival of patients with hepatitis B virus (HBV)-related cirrhosis. Therefore, we aimed to explore the difference in the mortality of HBV-related patients with liver cirrhosis (LC) based on the level of vWF-Ag and to evaluate whether the addition of vWF-Ag can improve the risk prediction ability of the MELD scoring system.

## 2. Materials and Methods

### 2.1. Patients

**Figure 1:**
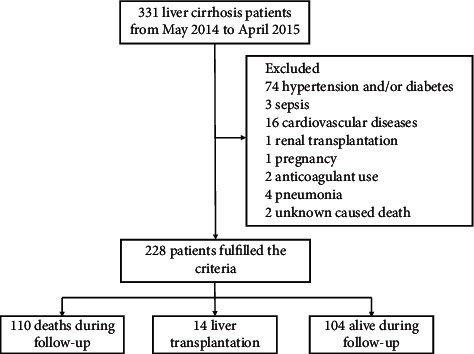
Flowchart of patient selection. 228 patients from 331 patients with liver cirrhosis (LC) were enrolled according to the criteria in this study, which were divided into three groups including deaths during follow-up, liver transplantation, and alive during follow-up.

This retrospective study consisted of 228 consecutive LC patients who visited the First Affiliated Hospital of Zhejiang University School of Medicine from May 2014 to April 2015. Liver cirrhosis was confirmed by either histology or unequivocal clinical and radiological findings. A diagnosis of ascites, oesophageal varices, spontaneous bacterial peritonitis (SBP), hepatic encephalopathy (HE), or hepatorenal syndrome was made according to the previously described criteria [24]. All LC patients were enrolled on the first day of hospitalization. They had not received treatment for complications related to cirrhosis, had not received a transfusion of fresh frozen plasma and platelets, and had not used vitamin K, warfarin, or other drugs affecting coagulation function within three months before admission. Patients with chronic hypertension, diabetes, coagulopathies, sepsis, cardiovascular diseases, anticoagulant or corticosteroid use, renal transplantation, HIV infection, pregnancy, and some related acute or chronic inflammation from nonhepatitis diseases, such as pneumonia and urinary inflammation, were excluded from the study. The patient selection process is shown in Figure 1. The study was approved by the Ethics Committee of the First Affiliated Hospital, Zhejiang University School of Medicine and was conducted according to the principles of the Declaration of Helsinki.

### 2.2. Sample and Data Collection

Venous blood samples were collected from all participants in the early morning after overnight fasting prior to any therapeutic procedure. One tube with 0.109 M trisodium citrate (9:1 v/v) was used for the measurement of D-dimer, fibrinogen, and vWF-Ag. Blood samples were immediately centrifuged at 1500 ×g for 10 min at 4°C to obtain plasma. A second tube without any anticoagulant was used to collect blood samples, and then the blood samples were allowed to clot to isolate serum after centrifugation at 3000 ×g for 10 min at 4°C. All plasma and serum samples were aliquoted and stored at −80°C until analysis.

Blood parameters included liver and kidney function indicators and coagulation parameters were obtained from electronic medical records. The level of vWF-Ag was measured using commercial enzyme-linked immunosorbent assay kits (R&D Systems Inc., Minneapolis, MN, USA) according to the manufacturer's instructions.

### 2.3. Statistical Analysis

Continuous variables were expressed as medians and interquartile range 25–75% (IQR) and compared with *t*-tests or the Mann–Whitney *U* test, and categorical variables were compared with the chi-squared test or Fisher's exact test and expressed as percentages. Patients were followed up from the day they enrolled in this study until liver transplantation or death occurred or until the last date of follow-up in December 2019. The endpoint of this study was defined as transplant-free survival, and the duration of follow-up was defined as the time until liver transplantation or death. The Kaplan–Meier method was used to evaluate the cumulative transplant-free survival probability and compared with a log-rank test in differences between groups. Hazard ratios (HRs) were calculated by the Cox regression model. Variables with *P* < 0.10 in the univariate analysis were included in the stepwise Cox multivariate regression model to assess the independence of predictors. Considering that patients with liver transplantation had a competing risk of death, the Fine and Gray regression model was used to assess the association between risk factors and patient death [25]. Furthermore, the receiver operating characteristic (ROC) analysis was performed to assess the potential discriminatory variables for transplant-free mortality. Youden's index was used to determine the optimal vWF-Ag cutoff level for distinguishing high-risk patients from low-risk patients. In addition, the generalized additive models with smoothing splines were used to determine whether vWF-Ag as a continuous parameter has a nonlinear effect on the risk of transplant-free mortality. To further maximize the predictive potential of identifying transplant-free mortality, we aimed to determine the potential value of including the vWF-Ag in the MELD scoring system. DeLong's method was used to compare the difference in AUCs between the two models [26]. To evaluate the robustness of the results, a time-dependent ROC curve was used to assess the predictive power of the MELD score and vWF-Ag level for the outcome. Statistical analyses were performed using R version 3.6.1 (R Foundation, Vienna, Austria). A two-sided *P* < 0.05 was considered statistically significant.

## 3. Results

### 3.1. Patient Characteristics

**Figure 2:**
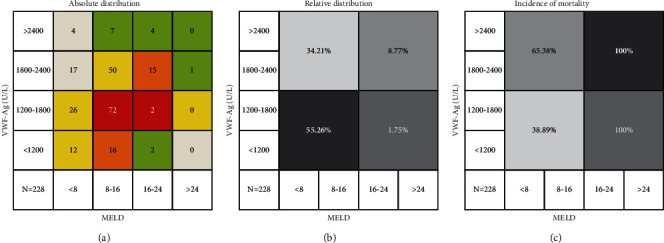
Association of vWF-Ag and MELD in patients with cirrhosis. (a) Absolute distribution of patients in regard to their vWF-Ag levels and MELD; (b) the relative distribution of patients in regard to their vWF-Ag levels and MELD; (c) the transplant-free mortality of patients with liver cirrhosis during follow-up in regard to their vWF-Ag level and MELD.

A total of 228 patients were enrolled in this study. The characteristics of the patients are listed in Table 1. Of the 228 patients, 76 (33.3%) were female, and the median age of the patients was 56 years (IQR 40–75 years). A total of 140 patients had vWF-Ag levels <1925 U/L, while 88 patients had values ≥1925 U/L, which was the optimal cutoff level for transplant-free mortality as determined by Youden's index. Patients with a high level of vWF-Ag had more advanced liver disease and a higher median MELD score (10.84 vs. 9.41 points; *P* < 0.001) and higher components of the MELD score (bilirubin and INR). This group also had more patients with stage C disease according to the Child–Turcotte–Pugh (CTP) score (28.4% vs. 19.3%), but this difference was not significant. Obviously, there were fewer patients with type O blood in the vWF-Ag low group (20 [22.73%] vs. 64 [45.71%], *P* < 0.001). During a median follow-up of 30.23 months (IQR 3.09–44.25 months), 124 patients (54.4%) reached the endpoint of this study, 14 (6.1%) of whom underwent liver transplantation, and 112 (49.1%) patients died. The vWF-Ag level was significantly higher in patients with complications of portal vein thrombosis (PVT), SBP, and ascites (Figure 2).

### 3.2. Association between the Level of vWF-Ag and the MELD Score and Transplant-Free Mortality

Initially, we explored the differences in the MELD score and vWF-Ag levels between patients who survived and died during follow-up. Indeed, patients who died or underwent liver transplantation showed an elevated MELD score (median MELD score, no mortality = 8.69; median MELD score, mortality = 11.44, *P* < 0.001). Similar results were observed for the vWF-Ag level (median vWF-Ag level, no mortality = 1516 U/L; median vWF-Ag level, mortality = 1957 U/L, *P* < 0.001). Surprisingly, during the follow-up, the vWF-Ag level and MELD score had the comparable predictive potential for transplant-free survival (area under the curve [AUC], vWF-Ag = 0.71; AUC, MELD = 0.73).

**Figure 3:**
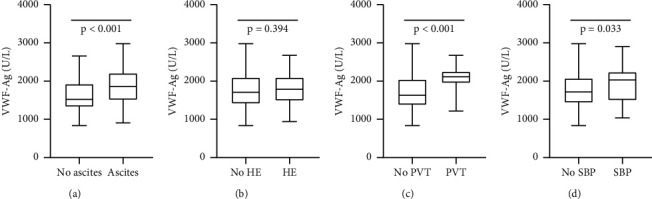
Comparing vWF-Ag levels between patients with different complications. (a)vWF-Ag levels between patients with or without ascites; (b) vWF-Ag levels between patients with or without hepatic encephalopathy; (c) vWF-Ag levels between patients with or without portal vein thrombosis (PVT); (d) vWF-Ag levels between patients with or without spontaneous bacterial peritonitis (SBP).

The comparative analysis of the vWF-Ag level and MELD score revealed a linear correlation between them. It was found that most patients had low vWF-Ag levels and low MELD scores, and a clear difference between patients with high vWF-Ag levels and MELD scores and those with low vWF-Ag levels and MELD scores was observed in regard to transplant-free mortality (Figure 3).

In the univariate analyses, the vWF-Ag level (*P* < 0.001), MELD score (*P* < 0.001), Child–Turcotte–Pugh score (*P* < 0.001), C-reactive protein level (*P* = 0.003), D-dimer level (*P* = 0.025), and aspartate aminotransferase level (*P* = 0.045) were associated with transplant-free mortality. In a stepwise multivariate Cox regression analysis, the vWF-Ag level, Child–Turcotte–Pugh score, MELD score, and C-reactive protein level were found to be independent variables and included in the model. As listed in Table 2, the vWF-Ag level (HR = 1.10/per 100 U/L) and MELD score (HR = 1.09) were significantly and independently associated with transplant-free mortality. When considering patients undergoing liver transplantation as a competing event, a competing risk analysis was used to assess the risk factors for death, and similar results were found (vWF-Ag: HR = 1.11 [95% CI, 1.06, 1.16]; *p* < 0.001, Table 3).

**Figure 4:**
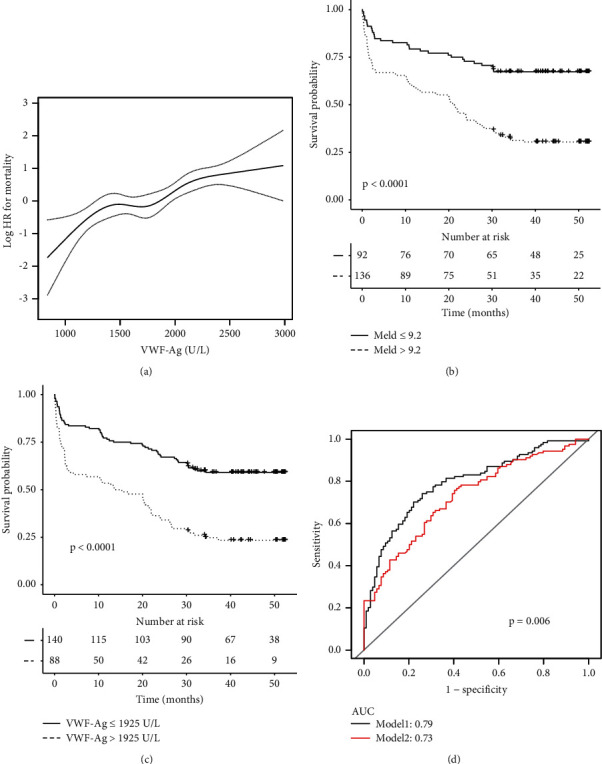
vWF-Ag allowing additional risk stratification on patients with liver cirrhosis during follow-up. (a) Continuous risk for transplant-free mortality (HR) calculated for vWF-Ag. (b) The cumulative probability of transplant-free mortality compared with a vWF-Ag cutoff at 1925 U/L; (c) The cumulative probability of transplant-free mortality compared with a MELD cutoff at 9.2 points; (d) VWF-Ag being able to increase the area under the curve (AUC) of MELD alone for prediction of transplant-free mortality on the patients with liver cirrhosis during follow-up.

An association was also observed between the vWF-Ag level and transplant-free mortality using Cox proportional hazards models adjusted for baseline covariates (Figure 4(a)). Using Youden's index, the optimal cutoff value for the vWF-Ag level was 1925 U/mL, which had a sensitivity of 0.80 and specificity of 0.54. Subsequently, the cohort was divided into two groups based on a high vWF-Ag level (≥1925 U/mL) and low vWF-Ag level (<1925 U/mL). Patients in the high-risk group were found to have a significantly higher incidence of mortality (76.1% vs. 40.7%, *P* < 0.001), which was comparable to that of patients in the high-risk group according to the MELD score cutoff value (69.1% vs. 32.6%, *P* < 0.001). Subsequent analysis found that the cutoff for vWF-Ag (1925 U/mL) had a similar hazard ratio (HR) for transplant-free mortality as the MELD score cutoff (9.2 points) (vWF-Ag, HR = 2.40, 95% CI [1.67, 3.43], *P* < 0.001; MELD, HR = 2.52, 95% CI [1.66, 3.82], *P* < 0.001, Figures 4(b) and 4(c)).

### 3.3. vWF-Ag Increases the Ability of MELD to Predict Transplant-Free Mortality

**Figure 5:**
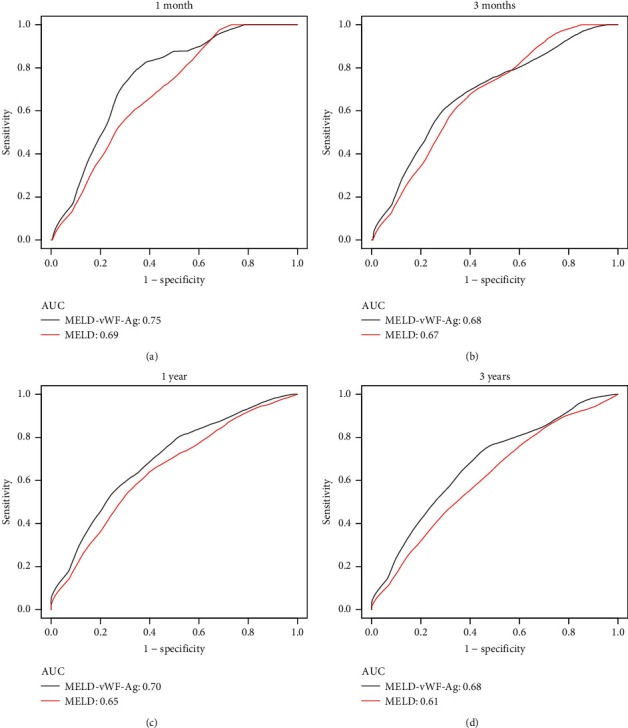
Time-dependent receiver operating characteristic (ROC) of MELD and MELD-vWF-Ag at 1 month, 3 months, 1 year, and 3 years for transplant-free mortality. (a) AUC of MELD and MELD-vWF-Ag at 1 month for transplant-free mortality; (b) AUC of MELD and MELD-vWF-Ag at 3 month for transplant-free mortality; (c) AUC of MELD and MELD-vWF-Ag at 1 year for transplant-free mortality; (d) AUC of MELD and MELD-vWF-Ag at 3 year for transplant-free mortality.

ROC curve analysis was performed, and the results revealed that incorporating the vWF-Ag level improved the discriminatory potential of MELD in determining transplant-free mortality in patients with cirrhosis (AUC, MELD = 0.73 [95% CI, 0.66–0.79]; AUC, MELD-vWF-Ag = 0.79 [95% CI, 0.73–0.85], *P* = 0.006, Figure 4(d)). Based on the results of ROC curve analysis, the optimal cutoff value for MELD-vWF-Ag yielded a sensitivity of 74.2% and a specificity of 74.0%. Considering that the active coagulation process in patients with PVT may alter the circulating vWF:Ag levels in plasma; we performed a stratified analysis based on whether patients had PVT or not and found that vWF:Ag could distinguish the high-risk patients well regardless of thrombosis (Figures S1 A, C). The combination of vWF could significantly improve the predictive power of the MELD score for transplant-free mortality in both groups (Figure S1). Furthermore, to evaluate the robustness of the results, we further applied time-dependent ROC curves to analyse transplant-free mortality using the MELD score and vWF-Ag level at 1 month, 3 months, 1 year, and 3 years. The AUCs of MELD–vWF-Ag for transplant-free mortality were 0.75, 0.68, 0.70, and 0.68, respectively; the AUCs of MELD were lower than those of MELD-vWF-Ag (0.69, 0.67, 0.65, and 0.61) at 1 month, 3 months, 1 year, and 3 years, respectively (Figure 5).

## 4. Discussion

In the present study, the risk of patients with liver cirrhosis (LC) was associated with the level of vWF-Ag, and the risk of mortality increased gradually as the level of vWF-Ag increased. We also assessed the potential for vWF-Ag to be used to substratify patients regardless of their MELD score. Indeed, patients with cirrhosis who had a vWF-Ag level higher than 1925 U/L were found to have shorter survival during follow-up, regardless of their MELD score at the time of enrolment. The mortality rate in patients with a vWF-Ag level below 1925 U/L and a MELD score below 9.2 was 26.9% during the almost 52 months of follow‐up, while patients with vWF-Ag levels above 1925 U/L and a MELD score above 9.2 had a very high mortality rate—approximately 87.3%. vWF-Ag can increase the potential of the MELD score to predict transplant-free mortality, regardless of whether short-term or long-term transplant-free survival is being assessed.

Although the MELD score was originally established to predict the survival of patients receiving transjugular intrahepatic portosystemic shunts [7], it has become a core part of prognostic evaluation and liver allocation in many countries around the world [27]. Patients with liver cirrhosis comprise a heterogeneous population with different aetiologies, such as viral hepatitis, including hepatitis B and hepatitis C, alcohol-related liver disease, and autoimmune hepatitis. The predictive potential of the MELD score in determining the survival of these patients also differs, and patients with a viral aetiology are at a unique disadvantage [28]. Additionally, the MELD score may not predict the outcome of decompensated patients, who have lower MELD scores [7]. Furthermore, many patients deteriorate rapidly due to severe complications or other decompensation-related events, which may lead to death in a short time [6]. Therefore, there is an urgent need for a new, accurate, and practical tool that can assess and predict the short-term and long-term prognosis of highly heterogeneous patients. This tool can help clinicians conduct reasonable stratification for the observation and treatment of patients to avoid wasting valuable medical resources.

In patients with cirrhosis, endothelial dysfunction is considered a major determinant of the increased vascular tone in cirrhotic livers, which plays a major role in the pathogenesis and continued worsening of cirrhosis [29–34]. A previous study revealed that the haemostatic system dysfunction associated with cirrhosis and the potential clinical effects of this dysfunction are topics of increasing interest [35, 36]. Previous studies [37, 38] demonstrated a strong correlation between the vWF-Ag level and portal hypertension, and the clinically significant complication of portal hypertension was assessed by the gold standard of hepatic venous pressure gradient (HVPG), which increased by 3.3 mmHg per 100 vWF-Ag increase. Indeed, the vWF-Ag is a marker for endothelial dysfunction, and it is considered a key pathophysiologic driver of complications associated with portal hypertension in patients [39]. As a biomarker of endothelial function, several studies have reported that the vWF level is related to the progression of liver cirrhosis. Mandorfer et al. reported substantially higher mortality in the high-risk group (higher vWF/CRP) than in the low-risk group (lower vWF/CRP), and they speculated that the prognosis of 5-year survival could be efficiently discriminated based on the level of vWF/CRP [38]. La Mura et al. indicated that an increase in the level of vWF-Ag is considered the most prominent feature of haemostatic dysfunction in LC patients, and they revealed that increased levels of vWF-Ag and FVIII could predict the presence of ascites and varices and even mortality in patients [40].

Our results showed that mortality in patients was associated with the level of vWF-Ag, and the risk of mortality increased gradually as the vWF-Ag level increased. Consistent with the previously published results, our multivariate analysis also showed that the level of vWF-Ag was positively related to mortality in patients with cirrhosis. One possible explanation is that vWF-Ag participates in primary haemostasis as a procoagulant factor, so it is likely that increased levels of vWF-Ag in patients might contribute directly to hypercoagulability and thrombosis. Matsuyama et al. reported that increased vWF-Ag over decreased ADAMTS13 may contribute to the progression of liver injury and even the development of multiorgan failure due to microcirculatory disturbances in the hypercoagulability state in patients with alcoholic hepatitis [41]. In addition, another study revealed that advanced cirrhosis was characterized by increased thrombogenesis and that targeting hypercoagulability could improve the outcomes of cirrhosis patients in terms of reducing the risk of potentially life-threatening complications [40]. To assess whether vWF-Ag could serve as a predictive marker, ROC curve analysis was used to evaluate the association of vWF-Ag levels with mortality in the present study. The optimal cutoff of vWF-Ag was 1925 U/L, which showed acceptable sensitivity and specificity. During the almost 52-month follow-up, patients with vWF-Ag levels above the cutoff value had significantly higher mortality.

The strengths of the present study are as follows: our data suggest that the addition of vWF-Ag can improve the prediction of the MELD scoring system in terms of risk stratification for patients with cirrhosis. The transplant-free mortality rate of patients with a vWF-Ag level below 1925 U/L and a MELD score below 9.2 was 26.9% compared with 87.3% in patients with a vWF-Ag level above 1925 U/L and a MELD score above 9.2. Based on the Cox regression model, a newly developed MELD-vWF-Ag model was obtained, and ROC curve analysis showed that the incorporation of vWF-Ag into the MELD scoring system can increase the possibility of clinically relevant predictions of mortality in patients with liver cirrhosis. However, we recognize that there are also several limitations. First, our data were collected in a single centre in China and require further external or internal verification. Second, only haematological indicators such as vWF and INR were included; other coagulation-related indicators (including factor FIII and a disintegrin and metalloprotease with thrombospondin type 1 repeats, member 13) were lacking.

In conclusion, an elevated vWF-Ag level is independently associated with transplant-free mortality in HBV-related patients with liver cirrhosis. Furthermore, including vWF-Ag in the MELD scoring system can improve the ability to accurately predict mortality in patients with liver cirrhosis through ROC curve analysis. These results suggest that vWF-Ag inhibition therapy may be considered as part of the treatment plan for patients with liver cirrhosis and may aid clinicians in optimizing monitoring strategies.

## Figures and Tables

**Table 1:** Clinical characteristics of patients at the time of enrolment.

Variable	Overall (*N* = 228)	vWF-Ag <1925 U/mL (*N* = 140)	vWF-Ag ≥1925 U/mL (*N* = 88)	*P* value
vWF-Ag (U/mL)	1738 (1448–2102)	1502 (1257–1687)	2220 (2065–2306)	<0.001
Age (year)	56 (51–59)	55 (51–58)	56 (52–59)	0.172
Female	76 (33.3%)	40 (28.6%)	36 (40.9%)	0.054
BMI (kg/m^2^)	21.85 (20.33–23.44)	21.95 (20.71–23.53)	21.78 (19.79–23.32)	0.156
MELD	9.90 (7.90–12.96)	9.41 (7.42–12.27)	10.84 (9.04–15.00)	<0.001
CTP stage				0.171
Stage A	47 (20.6%)	33 (23.6%)	14 (15.9%)	
Stage B	129 (56.6%)	80 (57.1%)	49 (55.7%)	
Stage C	52 (22.8%)	27 (19.3%)	25 (28.4%)	
Laboratory test				
Albumin (g/L)	34.10 (31.20–35.62)	34.10 (31.40–35.42)	34.40 (30.78–35.90)	0.872
INR	1.24 (1.10–1.35)	1.20 (1.08–1.31)	1.29 (1.13–1.44)	0.006
ALT (U/L)	24 (18–39)	24 (18–37)	25 (17–42)	0.961
AST (U/L)	42 (28–60)	40 (28–51)	47 (28–66)	0.263
TB (umol/L)	35.00 (18.00–62.00)	34.50 (17.50–53.25)	36.50 (18.00–92.75)	0.003
TCH (mmol/L)	2.84 (2.26–3.48)	2.88 (2.29–3.56)	2.79 (2.24–3.42)	0.155
UN (mmol/L)	5.70 (4.20–7.30)	5.60 (4.38–7.00)	5.70 (3.98–7.60)	0.248
Creatinine (umol/L)	65 (56–81)	67 (57–82)	63 (54–76)	0.414
LDH (U/L)	186 (152–229)	189 (154–230)	185 (149–217)	0.976
Leucocytes (10^9^/L)	3.70 (2.50–5.93)	3.85 (2.30–6.10)	3.50 (2.60–5.30)	0.959
CRP (mg/L)	8.10 (2.60–20.72)	7.55 (2.45–19.28)	11.55 (3.25–20.85)	0.942
Hemoglobin (g/L)	102 (84–118)	104 (84–118)	95 (81–115)	0.308
D-dimer (*μ*g/L)	2282 (976–4902)	2171 (928–4564)	2888 (1153–5945)	0.056
PLT (10^9^/L)	63 (35–116)	63 (37–113)	63 (34–120)	0.663
Fibrinogen (g/L)	1.69 (1.11–2.26)	1.66 (1.12–2.46)	1.73 (1.08–2.11)	0.082
HBV DNA (log_10_ IU/mL)	7.44 (6.05–8.01)	7.44 (5.97–8.00)	7.40 (6.41–8.17)	0.551
O blood group	84 (36.84%)	64 (45.71%)	20 (22.73%)	<0.001

vWF-Ag, von Willebrand factor antigen; BMI, body mass index; CTP, Child–Turcotte–Pugh; INR, international normalized ratio; ALT, alanine aminotransferase; AST, aspartate aminotransferase; TB, total bilirubin; TCH, total cholesterol; UN, urea nitrogen LDH, lactic dehydrogenase; CRP, C-reactive protein; PLT, platelet count; HBV, hepatitis B virus.

**Table 2:** Univariate and multivariate analyses for transplant-free mortality in patients with liver cirrhosis.

Variable	Univariate analysis		Multivariate analysis	
	HR (95% CI)	*P* value	HR (95% CI)	*P* value
MELD	1.13 (1.09–1.16)	<0.001	1.09 (1.05–1.13)	<0.001
CTP stage				
Stage A	1 (reference)		1 (reference)	
Stage B	2.42 (1.37–4.29)	0.003	1.72 (0.96–3.10)	0.069
Stage C	3.67 (1.98–6.82)	<0.001	1.95 (0.99–3.85)	0.053
Female (vs. male)	1.07 (0.74–1.54)	0.734		
Age (year)	0.99 (0.96–1.02)	0.672		
BMI (kg/m^2^)	1.01 (0.95–1.08)	0.644		
vWF-Ag (100 U/mL)	1.12 (1.08–1.17)	<0.001	1.10 (1.05–1.14)	<0.001
ALT (U/L)	1 (1.00–1.00)	0.629		
AST (U/L)	1 (1.00–1.00)	0.045		
TB (umol/L)	0.91 (0.78–1.06)	0.232		
UN (mmol/L)	1.03 (0.99–1.08)	0.177		
LDH (U/L)	1 (1.00–1.00)	0.078		
Leucocytes (10^9^/L)	1.02 (0.98–1.06)	0.372		
CRP (mg/L)	1.01 (1.00–1.01)	0.003	1.005 (0.995–1.011)	0.121
Hemoglobin (g/L)	1 (1.00–1.01)	0.547		
D-dimer (*μ*g/L)	1 (1.00–1.00)	0.022		
PLT (10^9^/L)	1 (1.00–1.00)	0.073		
Fibrinogen (g/L)	1.07 (0.92–1.25)	0.385		
O blood group (vs. non-O)	0.56 (0.38–0.82)	0.003	0.74 (0.50–1.11)	0.145
HBV DNA (log_10_ IU/mL)	1.03 (0.96, 1.11)	0.353		

HR, hazard ratio; vWF-Ag, von Willebrand factor antigen; BMI, body mass index; CTP, Child–Turcotte–Pugh; ALT, alanine aminotransferase; AST, aspartate aminotransferase; TCH, total cholesterol; UN, urea nitrogen LDH, lactic dehydrogenase; CRP, C-reactive protein; PLT, platelet count; HBV, hepatitis B virus.

**Table 3:** Multivariate analysis for transplant-free mortality in competing risk model.

Variable	Multivariate analysis	
	SHR (95% CI)	*P* value
MELD	1.09 (1.05, 1.14)	<0.001
CTP stage		
Stage A	Reference	
Stage B	1.62 (0.88, 3.01)	0.124
Stage C	1.81 (0.88, 3.71)	0.106
vWF-Ag (100 U/mL)	1.10 (1.05, 1.15)	<0.001
CRP (mg/L)	1.01 (1.00, 1.01)	0.071
O blood group (vs. non-O)	0.79 (0.51, 1.23)	0.296

SHR, subdistribution hazard ratio; vWF-Ag, von Willebrand factor antigen; CRP, C-reactive protein.

## Data Availability

The data used to support the findings of this study are included within the article.

## Supplementary Materials

Supplementary Materials Figure S1. Area under the curve (AUC) of vWF-Ag and MELD-vWF-Ag for transplant-free mortality. (A) AUC of vWF-Ag for transplant-free mortality in patients without portal vein thrombosis (PVT); (B) AUC of MELD and vWF-Ag for transplant-free mortality in patients without PVT; (C) AUC of vWF-Ag for transplant-free mortality in patients with PVT; (D) AUC of MELD and vWF-Ag for transplant-free mortality in patients with PVT.
