# Supplementary material for: The Role of von Willebrand Factor Antigen in Predicting Survival of Patients with HBV-Related Cirrhosis
Source: Can J Gastroenterol Hepatol. 2022 Mar 22;2022:9035971. doi: 10.1155/2022/9035971 (PMC8964228; doi:10.1155/2022/9035971)
Supplement: Supplementary Materials — Figure S1. Area under the curve (AUC) of vWF-Ag and MELD-vWF-Ag for transplant-free mortality. (A) AUC of vWF-Ag for transplant-free mortality in patients without portal vein thrombosis (PVT); (B) AUC of MELD and vWF-Ag for transplant-free mortality in patients without PVT; (C) AUC of vWF-Ag for transplant-free mortality in patients with PVT; (D) AUC of MELD and vWF-Ag for transplant-free mortality in patients with PVT. [file 9035971.f1.docx]

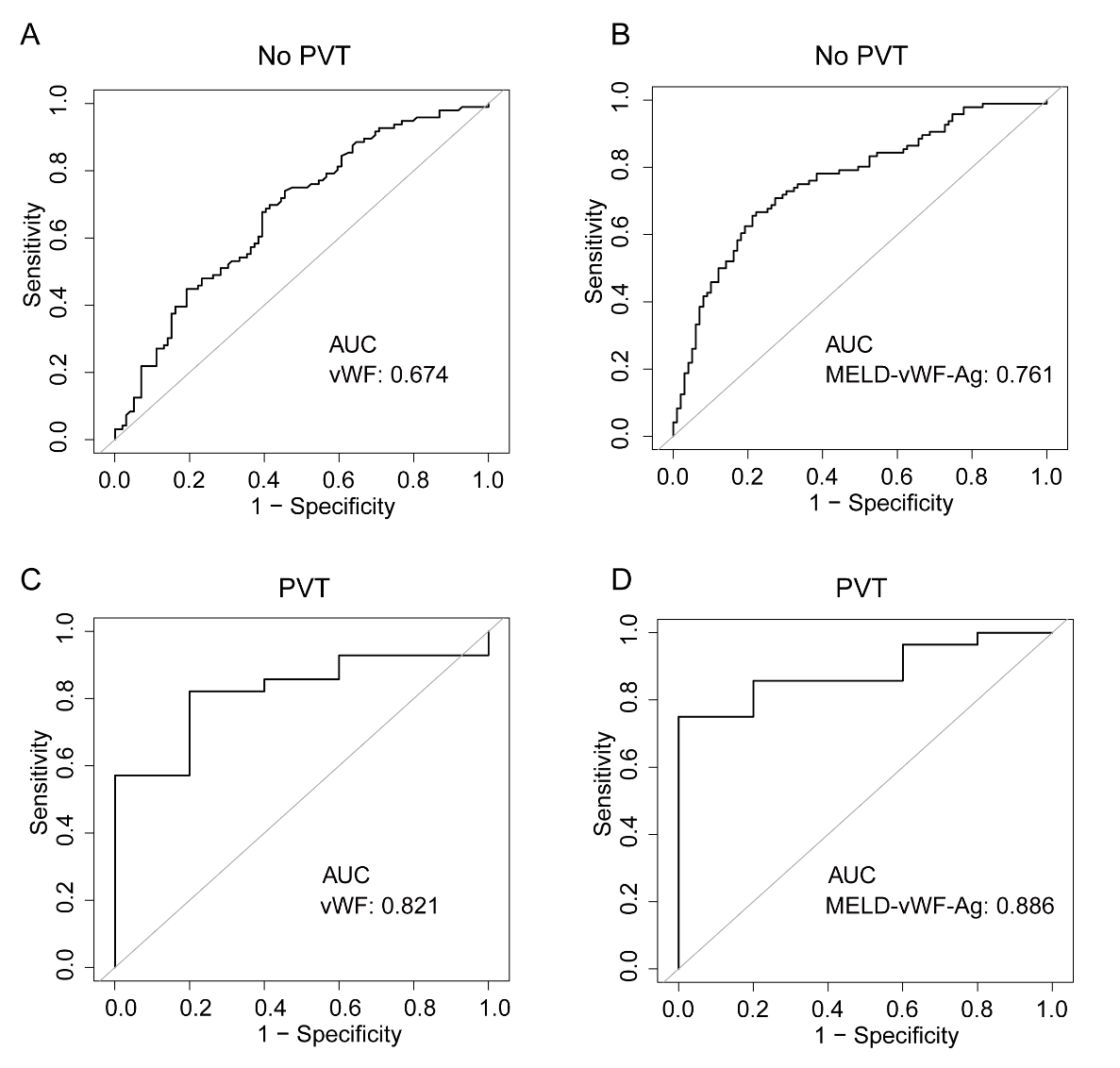


**Figure S1. Area under the curve (AUC) of vWF-Ag and MELD-vWF-Ag for transplant-free mortality.** (A) AUC of vWF-Ag for transplant-free mortality in patients without portal vein thrombosis (PVT); (B) AUC of MELD and vWF-Ag for transplant-free mortality in patients without PVT; (C) AUC of vWF-Ag for transplant-free mortality in patients with PVT; (D) AUC of MELD and vWF-Ag for transplant-free mortality in patients with PVT.
